# Health Prompts Affect Consideration of Health but Not Intertemporal Preferences While Promoting Healthier Food Choices

**DOI:** 10.3390/nu16101454

**Published:** 2024-05-12

**Authors:** Olivier Tuyizere, Christopher R. Gustafson, Devin J. Rose

**Affiliations:** 1 Department of Agricultural Economics, University of Nebraska-Lincoln, Lincoln, NE 68583, USA; 2 Department of Food Science and Technology, University of Nebraska-Lincoln, Lincoln, NE 68588, USA; drose3@unl.edu; 3 Department of Agronomy & Horticulture, University of Nebraska-Lincoln, Lincoln, NE 68583, USA

**Keywords:** point-of-decision prompt, active health consideration, health promotion, intertemporal preferences, nutrition, food choice

## Abstract

Diet-related diseases impact populations across the globe. While intertemporal preferences—a fundamental preference for the distribution of benefits across time—have been used to explain low-quality food choices, the recent literature proposes another cause: inattention to the future implications (or opportunity costs) of the options faced. Food choices tend to become habitual to conserve cognitive resources, rather than carefully modeling future health impacts. Both low discount rates for future benefits and attention to future health impacts predict healthier decisions. While intertemporal preferences are stable, attention may provide an opportunity to intervene in the decision process to promote healthier decisions. In this study, we test the impact of a simple message that highlights health during food choice on the healthiness of the foods chosen and on health consideration and intertemporal preferences. Our results show that actively considering health outcomes and lower discount rates lead to healthier food choices. We find that messaging increases the consideration of health outcomes during food choice but does not affect intertemporal preferences, suggesting that simple prompts may be an effective way to promote decisions balancing short- and long-term benefits by drawing attention to the overlooked opportunity costs of choices.

## 1. Introduction

Numerous seemingly minor daily choices that people face can have important impacts on their lives. Decisions about diet and exercise, whether to spend money on a desired item of clothing rather than saving the money for future use, or studying versus watching an extra episode of a favorite show seem insignificant at first glance, but making the same decision repeatedly may determine whether we are healthy later in life, have enough money for retirement, or achieve the academic degree that we desire. Individuals may fail to weigh the future costs of their repeated decisions [1,2]. Nevertheless, these minor daily choices influence important, long-term outcomes. Unhealthy food choices, infrequent physical activity, and low savings levels have long-term consequences for individuals and society. In fact, poor outcomes in life expectancy in the US and globally are attributable in part to these types of decisions [3,4].

Decision-makers in economic models of intertemporal choice weigh current and future options based on their preferences for the distribution of utility over time and choose the option that maximizes their wellbeing. While behavioral models allow for time-inconsistent preferences, both neoclassical and behavioral models of intertemporal choice model the decision-maker as considering current and future impacts [5]. Other disciplines, such as psychology, take different approaches to studying decisions with time-varying impacts. For instance, psychologists have developed the consideration of future consequences scale (CFC) to study differences in decision outcomes [6]. The CFC was designed to capture differences in individuals’ tendency to consider the future impacts of decisions when making choices. A systematic review and meta-analysis of research using tools such as the CFC found that future-oriented individuals are more likely to make decisions that are beneficial in the long run, such as exercising, saving more for retirement, and obtaining higher levels of education [7,8]. Future-oriented people are more likely to delay spending immediately and save for future expenditures [9,10]. Researchers have found that future orientation is causally related to healthy behaviors [11].

While the CFC was developed to measure an individual’s stable tendency to take the future into account, there is evidence that external factors influence cognitive processes. In the realm of health, for instance, research documents the influences of hunger [12] and choice timing [13] on the nutritional quality of foods selected. Simple e-mail reminders increase gym attendance, an effect that lasts beyond the end of the intervention [14,15], and health reminders increase the nutritional quality of purchases selected in brick-and-mortar stores [16,17] and in online settings [18,19]. In the financial domain, people exposed to questions about overdraft fees on surveys were less likely to accrue overdraft fees, an effect that persisted over multiple years [20]. Even in simple, acontextual research settings, explicitly highlighting the (delayed) opportunity cost of choosing a smaller, earlier monetary reward changes people’s choices, causing them to appear to value the future relatively more [21]. Therefore, external factors that direct attention to the health-promoting elements of choices may provide a useful tool to promote future wellbeing.

Food choice is a critical determinant of long-term outcomes. Diet-related diseases have been a primary contributing factor to the decrease in life expectancy and quality of life in the US in recent years [3,22], as well as significantly impacting health outcomes globally [4]. The food choice process is complex, yet it is a decision that individuals make daily. Since the 1990s, policies have been implemented in the US that require nutrition information and calorie labeling, but the obesity rate continues to rise [23]. A review of environmental nutrition interventions at the point of purchase recommends using interventions beyond food labeling [24]. Individuals find healthy eating challenging to achieve because it requires time and cognitive effort to identify and sustain healthy eating habits [25], which has led to a call for interventions that target automatic decision processes [26].

Our study examines the impact of an exogenous intervention that targets automatic decision processes by drawing attention to health outcomes during the food choice process. We examine the impact of this intervention on the consideration of health when facing food choices and on overall nutrition quality. A few studies have examined interventions that prompt individuals to consider long-term health benefits in the face of frequent decisions, without imposing restrictions on the individuals’ choices. For instance, the use of point-of-decision prompts has been found to significantly increase the use of stairs rather than an escalator/elevator [27,28,29]. Examples of point-of-decision prompts placed near stairs/elevators include “walking upstairs burns almost five times more calories than riding an elevator. Take the stairs”, “improve your waistline, use the stairs”, and “your heart needs exercise, use the stairs”, with footprints guiding people to the stairs. In the case of food choice, research conducted in physical and online food retail settings of prime or prompt messages presented at the point of decision have been found to increase healthier choices, including in high-risk populations, such as individuals with a high bodyweight and low-income minority populations [16,17,18,30].

Previous studies have found that actively considering the future implications of choices led to significantly healthier choices [1,31,32]. A study on the impact of exposure to health prompts during choice found an increased likelihood of considering health outcomes, although this study did not incorporate data on intertemporal preferences [32]. On the other hand, Tuyizere and Gustafson [2] found that both the active consideration of health during choice and low discounting rates led to healthier choices, although this study was cross-sectional and therefore could not attribute causality to these relationships.

In this study, we examine whether simple health prompts presented at the point of decision, which highlight health benefits—in this case, of dietary fiber—increase the active consideration of the health impacts of foods considered during the choice process. We examine the effect of the prompt in an online experiment on food choice along with an intertemporal financial choice task. The health prompt message focuses on dietary fiber, an under-consumed dietary component of public health concern, which may be driven by a lack of knowledge of the health benefits of fiber, leading people not to consider fiber during food choice [33,34,35]. The benefits of dietary fiber recognized by the FDA are (1) lowering blood glucose, (2) lowering cholesterol levels, (3) lowering blood pressure, (4) increasing the frequency of bowel movements, (5) increasing mineral absorption in the intestinal tract, and (6) reducing energy intake [36]. If a simple health message increases the proportion of people who actively think about future implications during food choice, it provides a valuable intervention tool at the point of purchase to increase attention towards healthier options without imposing restrictions on people’s options.

## 2. Materials and Methods

We conducted an online food choice experiment of 1005 US adults (≥19 years old) in August 2021. We developed the survey in Qualtrics (www.qualtrics.com, accessed 22 May 2021) and distributed it via Prolific (www.prolific.com, accessed 17 July 2021), an online survey recruitment platform. To participate in the experiment, individuals had to be at least 19 years of age and residents of the US. The food choice task included hypothetical food choices in three common food categories: breads, ready-to-eat breakfast cereals, and crackers. These categories were selected because products in these categories vary markedly in dietary fiber content, which was the topical focus of the message that we examined. Participants also answered questions about attention and cognition during the shopping experience, completed an intertemporal preferences task, in which they made choices among different amounts of money that would be received either immediately or in one month, and reported demographic information. In the survey, participants were reminded to consider other, real-world uses of their money when considering the products in the experiment, to reduce biases from hypothetical decisions. This is called a cheap talk script, which, as a meta-analysis has shown, is an effective means to reduce hypothetical bias [37]. A cheap talk script directs participants to consider where else they might use their money, which likely causes participants to consider the opportunity costs of choosing a product in the experiment.

To evaluate the effect of a health prompt on cognitive processes and preferences that promote healthier choices, participants were randomly assigned to one of two conditions: a control condition or a prompt condition. In the control condition, participants did not receive a health prompt message; however, all other instructions and questions were identical between the conditions. The health message displayed to participants in the prompt condition was “How can dietary fiber help you reach your health goals? While some benefits of fiber consumption are well known, dietary fiber has a number of surprising benefits. Benefits that are not widely known include that dietary fiber: (1) Reduces energy intake (by, for example, promoting feelings of fullness), which helps with weight loss (2) Lowers blood pressure (3) Increases absorption of important minerals (4) Lowers blood glucose (5) Lowers cholesterol levels. Choosing products with higher dietary fiber can help you meet your health goals!*”*.

Participants viewed cereal, bread, and cracker product categories sequentially. Participants faced 33 product alternatives in each product category. As in real-world retail settings—both in-store and online—participants had the ability to direct their attention to products, potentially resulting in the incomplete consideration of the full set of available products. In each product category, participants could view all available products or they could choose to view one of three product subsets, each containing 11 items.

Products were selected for inclusion in the experiment based on availability (i.e., those that are widely available and are not private store brands), as well as to provide products representing the breadth of nutritional quality available in the market. Subsets were inspired by sets observed in real-world retail settings (see Figure 2 in [18]). In each product category, the three sets of 11 items were categorized based on the Guiding Stars (GS) nutrition rating system. The GS rating system calculates a score for each product based on dietary components, such as the added sugars, added sodium, saturated fat, trans fat, vitamins, minerals, fiber, whole grains, omega 3, vitamins, and artificial colors contained in a product (see more information about the calculation of product scores at www.guidingstars.com, accessed 9 March 2020). The GS system rates products from 0 (low nutritional quality) to 3 (high nutritional quality) stars. The three subsets in each product category separated products into those with (1) zero GS, (2) one GS, and (3) two or three GS. The GS rating was not displayed to participants in the experiment; it was only used to represent the nutritional quality of the products.

To collect data on the factors that participants actively considered during the choice process, they answered a question after they had finished making all food choices. This question was, “In general, which of the following did you consider when making food choices today?” Responses to this question were offered in a check-all-that-apply format. The data of interest were captured by participants’ responses to the following items: “the impact of the foods on your/your family’s current health” and “the impact of the foods on your/your family’s health in the future”. Other items were included as decoys to mask the true items of interest. We collected these data from participants after they had made their choices, to avoid influencing their choices during the consideration process, reflecting an approach that has been used in other studies focused on capturing elements of the thought process during decision-making [38]. Finally, participants answered questions about intertemporal preferences and demographic variables. The intertemporal preference question featured a series of hypothetical choices between a sooner, smaller monetary amount and a larger, delayed amount, which has been used in previous studies [2]. The first choice featured the option to receive USD 1000 immediately or USD 1200 in one month. If the individual selected the delayed amount, they continued to the next question; otherwise, they were faced with a choice between USD 1000 immediately and USD 1300 in one month. If a participant again selected the smaller, earlier amount, they subsequently faced a choice between USD 1000 immediately and USD 1400 in one month. If they indicated a preference for USD 1000 immediately in each of these three choices, they were then asked about the amount that they would need to receive in one month to cause them to select the delayed amount. Participants who selected USD 1200 in the first choice were categorized as having a low discount rate, those who selected USD 1300 in the second choice were categorized as having a medium–low discount rate, those choosing USD 1400 were categorized as having a medium–high discount rate, and those who selected the earlier amount in each of the first three questions were categorized as having a high discount rate. To ensure that the participants paid attention, we asked a question in which they were asked to mark “added sugar” from a list of six options. Five participants who did not mark “added sugar” were excluded from the analysis.

We conducted the analysis using R Studio, v4.1.2 [39]. We used logistic regression to examine the impact of the prompt on the active consideration of health and multinomial logistic regression for the discount rate categories. In each case, the dependent variable (active consideration of health, discount rate) was regressed on the exposure to the prompt message. We examined the impact of these independent variables alone and in a second version in which we included demographic variables: sex, age, income, and education. We incorporated cluster robust standard errors at the individual level using the lmtest package [40].

To examine the nutritional quality of the foods chosen, we created a panel dataset of the food choices that every participant made, resulting in three rows per participant—one row for each food category. We conducted linear regression to examine the impact of the active consideration of health, discounting (low, medium–low, or medium–high, relative to an omitted “high discount rate” category), and prompt exposure on the Guiding Stars ratings of the foods chosen in experiment with and without demographic controls. We hypothesized that exposure to the health message would lead to healthier choices (measured in GS ratings), while also identifying significant impacts of the active consideration of health and discount rates.

The simple version of the linear regression model of GS and the consideration of health impacts, discounting, and prompts (excluding demographic characteristics) is as follows:

(1) $$GS_{ij}=\beta_{0}+\beta_{1}\left(H_{i}\right)+\beta_{2}\left(D_{i}\right)+\beta_{3}P_{i}+\in_{ij}.$$

Here, *GS_ij_* is the number of Guiding Stars chosen by individual *i* for product *j*, *H_i_* is the active consideration of health impacts (current and/or future) by individual *i*, *D_i_* is the discount rate category for individual *i*, *P_i_* is health prompt exposure for individual, *i* and $\in_{ij}$ is the error term. We report variables with coefficient estimates at (*p* < 0.05) as statistically significant. The study protocol was approved by the University of Nebraska–Lincoln’s institutional review board (#20201020721EX). All participants provided electronic consent before participating in the study.

## 3. Results

We report the summary statistics of the participant sample in Table 1. Over 70% of the participants were female. The mean age of the participants was slightly over 29 years. Approximately 56% had completed a bachelor’s degree or higher, and the average household income of participants was slightly over 75,000 US dollars.

Additionally, approximately half of the participants reported considering their health during food choice, while most participants selected the larger delayed monetary amount immediately. The average product selected by a participant in the study had slightly under one Guiding Star.

We report the results of a logistic regression to examine the impact of the prompt on the active consideration of health in Table 2. The results show the significant impact of exposure to the health message on the active consideration of health, both with and without the inclusion of demographic variables as controls. Specifically, exposure to the prompt message increased the likelihood that a participant actively considered the health impacts of the foods in the choice set by nearly 1.3 times in both models. We report the full results of Model 2 in Table 2, with demographic characteristics, in Supplementary Table S1. This result corresponds to our hypothesis that simple messages can direct attention to the attributes of choice alternatives.

In the second analysis, we evaluated whether exposure to the health prompt affected individuals’ discount rates. We conducted a multinomial logistic regression of the discount rates on the prompt (Table 3). There was no significant impact of the prompt message on the discounting rates among the participants in our research. We report the full results of the model with demographic characteristics in Supplementary Table S2.

The third analysis was a linear regression of the Guiding Stars ratings of the foods selected by participants (the dependent variable) on the active consideration of health, discount rate categories, and prompt exposure, to evaluate impacts on the healthiness of the participants’ product choices. We conducted this analysis in the simple form initially and then added demographic variables as a robustness check.

When demographic variables were not included in the model, the consideration of health outcomes led participants to choose products with 0.524 more GS per product, respectively, than those who did not consider health outcomes (Table 4). Individuals with low discount rates chose products with 0.178 more GS per product than impatient participants. Participants who received a health prompt message chose more nutritious products, equivalent to 0.133 GS per product, compared to those who were not exposed to the message. Similar results were found when demographic variables were included in the model. We report the full results of the model with demographic characteristics in Supplementary Table S3.

## 4. Discussion

Our findings show that exposure to health prompts increased the likelihood that people actively considered the health outcomes during food choice and led them to select foods with higher nutritional quality. Studies have found that people who discount the future heavily are more likely to have lower-quality diets and engage in other behaviors that put them at risk for obesity and other poor health outcomes [41,42,43,44,45,46,47]. A smaller body of evidence suggests that asymmetric attention to the immediate vs. future opportunity costs of choices may play an important role in determining choices with intertemporal components [1,2,21,32]. This evidence suggests that attention is more naturally paid to immediate opportunity costs, yielding decisions that appear to represent preferences that heavily discount the future but that may instead represent blindness to future opportunity costs. In our study, the evidence of the impact of the prompt on the consideration of health and discounting choices suggests that health prompts work by directing attention to the attributes of the products faced, but do not change people’s preferences for the temporal distribution of the costs and benefits. The finding that simple prompts do not change intertemporal preferences is consistent with research showing that changing an individual’s discount rate—so that they are more patient and thus more likely to make choices that provide greater long-term benefits—requires intensive educational intervention [48] or shifts in one’s beliefs about one’s connection to one’s future self [49,50]. Therefore, our results reveal that simple efforts that prompt the active consideration of the health impacts during food choice may be an effective complement to more intensive interventions that aim to alter discount rates. These findings also concur with a recent study showing that attention to a health prompt message increases the consideration of future health during food choice [32].

Episodic future thinking (EFT) is a concept that has been used in psychology, cognitive development, and child development research to help individuals to envision future events so that they actively think through the possible future outcomes of choices that they face, helping them to establish pathways to attain these outcomes [51,52]. EFT helps a person to pre-experience events so that they conceptualize their feelings and the actions to be taken to achieve their future self’s goals. Evidence shows that EFT decreases the tendency to discount delayed gratification in intertemporal choice tasks [53]. In the absence of EFT (or other interventions), future rewards tend to be devalued, while immediate rewards are likely to be overestimated, leading to shortsighted choices [54]. EFT encourages positive health practices in intertemporal choice scenarios [55], such as limiting snacking [56], reducing impulsive eating and calorie intake in overweight or obese individuals and among individuals with a varying overweight status [57,58], and reducing cheating by inducing future orientation with a focus on one’s ideal self [59]. Evidence from the literature on EFT supports our findings that decisions incorporating the active consideration of the future impacts of choice alternatives faced now lead to better choices. While our results suggest that brief, targeted messaging improves the quality of choices, future research may investigate whether EFT combined with interventions prompting the active consideration of health outcomes may be even more effective. Combined interventions may help people with obesity or overweight to lose weight by vividly imagining their desired future (EFT), providing motivation to take action, paired with targeted prompts that make this imagined future salient in food choice settings.

Our study found that participants exposed to health prompt messages considered health impacts more and chose more nutritious food products than those not exposed to prompts. The increasing sophistication and power of technology may be used to help people to think more about the future implications of their food choices, by using health prompt messages about the benefits of food—for instance, sending a message that reminds them of the benefits of fiber or under-consumed nutrients when they are making choices in the store or online. Health professionals may use this concept to send health message reminders to their clients about the future impacts or health benefits of foods that may significantly increase the quality of their eating habits. A podcast-based educational intervention during grocery shopping led shoppers to buy more ω-3-rich seafood items when given podcasts about the types, food sources, and health benefits of ω-3 fatty acids at the point of purchase throughout a 6-month intervention and 6 months post-intervention [60,61]. This study shows that interventions as messages or other interventions highlighting health benefits at the point of purchase may help individuals to make healthier choices. While point-of-decision actions are a key marketing strategy—for instance, the profusion of gluten-free labeling on products once an interest in gluten-free foods emerged in the general public [62]—promoting consideration of health at the point of decision has not been widely implemented.

It is also important to consider the audience and the complexity of the messaging. An inverse relationship between educational attainment and obesity exists, especially in high-income countries [63], and too little knowledge can be a barrier to comprehension [64]. We used a brief, simple message, as these have been found to more effectively communicate health information to individuals with low or high health knowledge [65] and to have a greater impact on increasing healthy choices [66].

The findings of our study have some broader implications. Identifying the active consideration of health outcomes during food choice—and showing that a simple educational prompt can increase the active consideration of health—provides further insights into the impact of exogenous cues in the choice environment. Studies on prompts have found that exposure to prompts during food choice increases healthy behaviors [66,67]. Related research on physical activity found that sending reminders to gym members increased attendance and had effects that lasted beyond the end of the intervention [14,15]. While previous research has documented that prompts change multiple choice process behaviors—such as the use of nutrition information and the sets of products considered during choice [68]—impacts on cognition have not been studied in complex choice environments. In an fMRI study, participants made healthier decisions in a food choice task when they were exposed to prompts directing their attention to their health [69], resulting in neural and behavioral patterns that resembled those of successful dieters [70].

Our study has limitations that need to be addressed in further work. The food choice task in this study was hypothetical. Participants did not pay for and receive their food selections as they would in a real-world food retail outlet. Our experimental task was hypothetical so that we could collect a large set of data from participants across the US. While we used established methods to minimize the risk of hypothetical bias, by including a cheap talk script that directed participants to approach the choice task as if they would make an actual transaction [37], observing real, binding choices would provide stronger evidence about the effect of consideration, discounting, and prompts. However, separate elements of the study are known to have impacts in real choice settings, including intertemporal preferences [71] and the effect of prompts on choices in food choice [16,17].

A second limitation is the potential exclusion of people who possess such a regular habit of making healthy food selections that they no longer actively consider their health during choice, but rather habitually choose from a small subset of products that meet previously establish health criteria. We asked the participants about the factors that they actively considered during the choice process, to capture those who had actively considered their health, and—even more importantly—to allow us to detect changes in the active consideration of health. Participants who habitually made healthy choices may not have reported actively considering their health during choice because healthy choices had become habitual for them. In this case, our finding that those who actively considered the health outcomes made significantly healthier choices may underestimate the value of establishing healthy habits, because the no-consideration group would include anyone who habitually made healthy choices.

A third limitation of the study is that the sample of participants was not representative of the adult US population. Our sample was predominantly female, younger, and more highly educated than the adult US population. Women tend to be more likely to be primary shoppers, so it is not surprising that the sample had a higher percentage of women than men and, in fact, this was similar to the percentage of women in previous studies that targeted primary shoppers [33,34,72,73]. Individuals with higher education levels tend to have better outcomes [63,74], while older individuals pay greater attention to certain aspects of food choices [33]. However, since we examined behavior in an experiment with randomization to condition and control for demographic characteristics, the non-representative nature of the sample does not threaten the validity of our results.

This study contributes to a growing body of research suggesting that the active consideration of health outcomes and exposure to health prompt messages promote healthier food choices. These methods may provide a simple, low-cost approach to stimulate the consideration of the often-overlooked health impacts of food choices. While additional research is necessary, these methods may complement other intervention approaches, such as EFT, so that an individual can both establish a desired future outcome and actively consider the implications of the choices that they face for this desired future outcome. Our findings show that exogenous factors such as simple messages intended to bring attention to the consideration of future opportunity costs during choice are an important tool to engender the active consideration of future impacts during the decision process. This approach may help to align choices with long-term preferences in other domains in which people face tradeoffs between short- and long-term benefits, such as promoting savings for the future, making environmentally sustainable choices, and others.

## 5. Conclusions

In this research, we investigate the potential of targeting attention to future health impacts as a intervention tool to tackle behaviors subject to habitual decision-making, which is a critical barrier to the attainment of healthy outcomes [26]. While intertemporal preferences have long been used to model long-term health outcomes, preferences are difficult to change. Attention, on the other hand, is variable and can be influenced by environmental stimuli. This study shows that a simple message delivered at a critical time—shortly before an individual begins to make a food choice—can promote healthier choices by increasing the consideration of health outcomes in the alternatives that the individual faces and by directly inspiring healthier choices.

## Figures and Tables

**Table 1:** Demographic characteristics of study participants (*N* = 1005).

Variables	Mean	SD
Female (%)	72.9	
Age (years)	29.1	26.0
Education (%):		
Advanced degree (Master’s degree or higher)	26.1	
Bachelor’s degree	30.0	
Associate degree or some college	27.2	
High school/G.E.D.	15.5	
Less than high school	0.7	
Income (USD 1000s)	75.5	70.0
Actively consider health (%)	49.7	
Low discount rate (%)	75.8	
Medium–low discount rate (%)	6.4	
Medium–high discount rate (%)	5.6	
High discount rate (%)	12.1	
Guiding Stars	0.96	0.92

**Table 2:** Logistic regression of the effect of the health prompt message on the active consideration of health.

	(1) OR (95% CI)	(2) OR (95% CI)
Health prompt	1.28 (1.11, 1.48)	1.29 (1.10, 1.50)
Constant	0.87 (0.79, 0.97)	0.00 (0, 4.8 × 10^11^)
Demographic variables	No	Yes

Notes: OR = odds ratio; 95% CI = 95% confidence interval.

**Table 3:** Multinomial logistic regression for the effect of the health prompt message on the discount rate categories (reference: high discount rate category).

Odds Ratio (95% Confidence Interval)						
	Med–High Discount	Med–Low Discount	Low Discount	Med–High Discount	Med–Low Discount	Low Discount
Health prompt	0.71 (0.49, 1.03)	1.20 (0.84, 1.72)	0.90 (0.72, 1.13)	0.76 (0.51, 1.13)	1.15 (0.79, 1.68)	0.88 (0.68, 1.14)
Constant	0.55 (0.42, 0.71)	0.48 (0.36, 0.62)	6.58 (5.59, 7.74)	0.09 (0.02, 0.39)	0.10 (0.02, 0.44)	57,197 (17,142, 190,849)
Demographic variables		No			Yes	

Notes: Reported values are the estimated coefficients, with standard errors in parentheses.

**Table 4:** Linear regression of active consideration impact, discount rates, and prompt exposure on the Guiding Stars ratings of the foods chosen.

	Coef. (SE) (1)	Coef. (SE) (2)
Consideration of health	0.524 *** (0.033)	0.476 *** (0.034)
Low discount rate	0.178 *** (0.051)	0.236 *** (0.057)
Med–low discount rate	−0.045 (0.079)	0.013 (0.081)
Med–high discount rate	0.005 (0.083)	0.045 (0.086)
Prompt	0.133 *** (0.033)	0.136 *** (0.033)
Constant	0.497 *** (0.055)	0.220 (0.541)
Demographic variables	No	Yes
Observations	2905	2905
R^2^	0.091	0.108
Adjusted R^2^	0.089	0.100

Notes: Data were collected in the experiment/survey. *** = *p* < 0.001.

## Data Availability

The data presented in this study are available on request from the corresponding author. The data are not publicly available due to privacy.

## Supplementary Materials

The following supporting information can be downloaded at: https://www.mdpi.com/article/10.3390/nu16101454/s1, Table S1: Logistic regression of the effect of the health prompt message on the active consideration of health with demographic control variables; Table S2: Multinomial logistic regression for the effect of the health prompt message on the discount rate categories with demographic control variables; Table S3:Linear regression of active consideration impact, discount rates, and prompt exposure on the Guiding Stars ratings of the foods chosen with demographic control variables.
